# Commercial Automated Insulin Delivery Systems in Type 1 Diabetes Pregnancy: Protocol for a Mixed‐Methods Systematic Review and Meta‐Analysis of Glycaemic, Perinatal, Psychosocial and Implementation Outcomes

**DOI:** 10.1002/edm2.70309

**Published:** 2026-09-05

**Authors:** Jieli Li, Elahe Sheklabadi, Rebecca F. Goldstein, Ashley H. Ng, Helena Teede, Negar Naderpoor

**Affiliations:** ^1^ Monash Centre for Health Research and Implementation, Faculty of Medicine, Nursing and Health Sciences Monash University Melbourne Victoria Australia; ^2^ Diabetes and Endocrinology Unit Monash Health Melbourne Victoria Australia

**Keywords:** automated insulin delivery, hybrid closed loop, pregnancy, psychosocial outcomes, Type 1 diabetes

## Abstract

**Background:**

Commercial automated insulin delivery (AID) systems are increasingly used in pregnancies complicated by Type 1 diabetes, but the evidence is dispersed across trials, observational studies, case series and qualitative research. Existing syntheses have largely emphasised glycaemic efficacy and selected perinatal outcomes, with less attention to device platform, timing of initiation, psychosocial burden, clinician workload and implementation context.

**Aims:**

To describe the protocol for a mixed‐methods systematic review and meta‐analysis evaluating the effectiveness, safety, feasibility, acceptability and implementation of commercial AID systems in Type 1 diabetes pregnancy.

**Methods:**

MEDLINE, Embase, CINAHL, Scopus, Emcare, the Cochrane Library, CENTRAL and ClinicalTrials.gov will be searched from 1 January 2010, with an update search before final synthesis. Eligible evidence will include randomised and non‐randomised comparative studies, observational cohorts, case series, qualitative studies and mixed‐methods studies involving pregnant women with Type 1 diabetes using commercial AID systems, including off‐label pregnancy use. Two reviewers will independently screen, extract and appraise studies and inter‐rater agreement will be reported. Randomised and non‐randomised comparative evidence will be synthesised separately and, where studies are sufficiently comparable, meta‐analysed within study‐design and comparator strata. Quantitative outcomes will include pregnancy‐specific time in range, other continuous glucose monitoring (CGM) metrics, maternal and neonatal outcomes, diabetes‐specific adverse events, psychosocial measures, cost, resource use, feasibility, implementation and early postpartum outcomes. Qualitative evidence will be synthesised thematically and integrated with quantitative findings using a convergent segregated approach.

**Discussion:**

The review will clarify what is known about commercial AID use in Type 1 diabetes pregnancy and identify patient‐, device‐, clinician‐ and service‐level factors relevant to implementation.

**Trial Registration:**

This systematic review protocol is registered with PROSPERO: CRD420251025194

## Graphical abstract

This protocol outlines a mixed‐methods systematic review and meta‐analysis of commercial automated insulin delivery in Type 1 diabetes pregnancy, integrating clinical outcomes with women's and clinicians' experiences, feasibility, cost and implementation evidence to inform safe, acceptable and equitable pregnancy care.

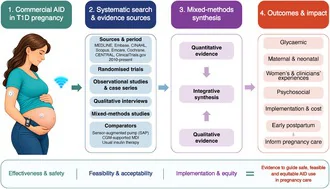

## 1 Introduction

Pregnancy complicated by Type 1 diabetes (T1D) presents one of the most stringent clinical settings for diabetes technology. Glycaemic targets are narrow, insulin requirements change rapidly and treatment decisions may influence both maternal and neonatal outcomes. Commercial automated insulin delivery (AID) systems are increasingly being used in this setting, but uptake varies substantially with regulatory approval, reimbursement, workforce capacity and local service models and no reliable global pregnancy‐specific estimate is available. As a regional benchmark, the 2025 National Pregnancy in Diabetes Audit for England and Wales recorded pregnancy‐specific CamAPS FX use at 28 weeks in 895 of 1995 Type 1 diabetes pregnancies with insulin method recorded (44.9%), with a further 240 (12.0%) using another hybrid closed‐loop system; because these data were collected only from May 2025, they may underestimate use [1]. This estimate is a regional benchmark within a publicly funded implementation context and should not be extrapolated to jurisdictions with different reimbursement arrangements. This variability creates a timely need to synthesise not only glycaemic and perinatal outcomes, but also the contextual factors relevant to safe, feasible and equitable implementation.

Women with Type 1 diabetes remain at increased risk of adverse pregnancy outcomes, including pre‐eclampsia, preterm birth, caesarean delivery, macrosomia, neonatal hypoglycaemia and admission to neonatal intensive care units [2, 3]. Diabetes‐specific maternal risks include severe hypoglycaemia, diabetic ketoacidosis and progression of microvascular complications, particularly retinopathy and renal disease; neuropathic complications may also affect pregnancy care where present [4, 5, 6, 7]. Large national and international studies continue to report higher rates of large‐for‐gestational‐age birth, preterm delivery and neonatal intensive care admission compared with the general obstetric population, despite specialist care and increasing use of diabetes technologies [2, 3, 5]. Achieving pregnancy‐specific glycaemic targets is central to reducing these risks, but remains difficult because insulin sensitivity changes across gestation, glycaemic variability increases and intensive self‐management imposes substantial burden on women, families and care teams [4, 8, 9, 10, 11]. Current guidelines therefore emphasise preconception optimisation and maintenance of glucose and glycated haemoglobin (HbA1c) levels as close to the physiological range as can be achieved safely [4, 5, 7].

Continuous glucose monitoring (CGM) has transformed the assessment of glycaemic exposure in Type 1 diabetes pregnancy and has established time in the pregnancy‐specific target range (TIR) of 3.5–7.8 mmol/L (63–140 mg/dL) as a clinically meaningful metric [8, 9, 10, 11]. Randomised and observational studies, including CONCEPTT and large cohort analyses, show that higher pregnancy‐specific TIR and lower glycaemic variability are associated with lower risks of large‐for‐gestational‐age birth, neonatal hypoglycaemia and neonatal intensive care admission [9, 10, 11]. However, current approaches, including sensor‐augmented pump therapy, continuous subcutaneous insulin infusion with CGM and CGM‐supported multiple daily injections, still rely substantially on user‐ and clinician‐directed insulin adjustment [8, 9, 10, 11]. Clinically important hyperglycaemia and hypoglycaemia can therefore persist, particularly as insulin requirements rise and change rapidly during pregnancy.

AID systems, most commonly delivered through hybrid closed‐loop platforms, combine CGM, insulin pump therapy and embedded algorithms that modulate insulin delivery in response to sensor glucose levels [12, 13, 14]. In non‐pregnant populations, randomised trials and meta‐analyses show that AID can increase TIR and reduce time below range compared with sensor‐augmented pump therapy or CGM‐supported multiple daily injections, without clear increases in severe hypoglycaemia or diabetic ketoacidosis [13]. Several commercial systems have now been used during pregnancy, including CamAPS FX, MiniMed systems, Tandem Control‐IQ and other commercial platforms [12, 14, 15, 16, 17, 18, 19, 20, 21, 22, 23, 24]. Regulatory status is jurisdiction‐ and time‐specific. CamAPS FX has an explicit pregnancy indication in the US Food and Drug Administration clearance when paired with a CGM suitable for pregnancy, whereas other platforms may be used off‐label or within pregnancy‐specific clinical evaluation depending on the jurisdiction [25]. Other platform‐jurisdiction combinations may be approved, under evaluation or used off‐label as labelling evolves. The review will therefore extract device, jurisdiction, study period and regulatory status rather than assign a single global approval category. Pregnancy is not simply an extension of non‐pregnant Type 1 diabetes care: many commercial algorithms were not originally designed around pregnancy‐specific glucose targets. This raises clinical questions about device suitability, target settings, timing of initiation, clinician oversight, intrapartum use and adaptation to rapidly changing insulin requirements [12, 17, 19].

Pregnancy‐specific trials and real‐world studies have advanced the field, but they do not yet provide a simple or uniform answer. Randomised trials, including AiDAPT with CamAPS FX, CRISTAL with MiniMed 780G and CIRCUIT, have reported improved glycaemic outcomes in selected settings, but they differ in device platform, comparator, algorithm target, timing of initiation and support model [15, 17, 19]. Observational cohorts and case series add important real‐world information, but vary in sample size, baseline technology use, gestational timing, outcome definitions and reporting of automation exposure [16, 17, 18, 20, 21, 22]. As a result, commercial AID in pregnancy should not be interpreted as a single homogeneous intervention. The clinical meaning of the evidence depends on which system is used, when it is started, how it is supported and whether outcomes are assessed across glycaemic, maternal, neonatal and implementation domains [18, 23].

Three recent reviews address important but narrower aspects of this field [12, 13, 24]. One is a clinical overview of AID in pregnancy, one evaluates AID across the broader Type 1 diabetes population and one meta‐analyses randomised pregnancy trials. The present review adds a prespecified synthesis of commercial systems across randomised, non‐randomised, single‐arm, qualitative and mixed‐methods evidence; device‐ and comparator‐specific analyses; timing of initiation and regulatory context; overnight and early postpartum glycaemic outcomes; diabetes‐specific safety outcomes; validated patient‐reported measures; cost, reimbursement and resource use; and formal integration of women's and clinicians' experiences with quantitative findings. Pregnancy‐specific TIR is retained as the clinically appropriate primary outcome rather than presented as a novel contribution. Access, equity and workload will be treated as descriptive and contextual domains; causal attribution will be made only where supported by an appropriate study design and analysis.

Taken together, the evidence base is clinically important, rapidly evolving and methodologically heterogeneous. A mixed‐methods review is therefore needed because quantitative and qualitative evidence answer complementary questions. Using a convergent segregated design, quantitative effectiveness and safety findings and qualitative experience and implementation findings will first be synthesised separately and then integrated through structured side‐by‐side comparison. This protocol describes a PRISMA‐P‐aligned review of commercial AID systems in Type 1 diabetes pregnancy. It aims to determine not only whether these systems improve outcomes, but which systems and comparators have been studied, when treatment is initiated, how outcomes vary by study design and care context and what patient‐, clinician‐, device‐, financing‐ and service‐level considerations are reported.

## 2 Review Questions

- What is the comparative effectiveness and safety of commercial AID systems, analysed by device, comparator and study design, for glycaemic, maternal, neonatal and early postpartum outcomes in pregnant women with Type 1 diabetes?
- What feasibility, acceptability, psychosocial, cost, reimbursement, resource‐use and workload outcomes are reported for pregnant women, families, clinicians and services using or supporting AID in Type 1 diabetes pregnancy?
- What patient‐, clinician‐, device‐, regulatory‐, financing‐ and service‐level factors are reported as barriers to or enablers of AID use in pregnancy care?

## 3 Inclusion Criteria

### 3.1 Participants

The review will consider studies involving pregnant women with T1D at any gestational age and parity. Mixed‐population studies will be included only when data for pregnant women with T1D are reported separately or can be obtained from authors.

### 3.2 Intervention

The quantitative component will consider commercial AID systems used during pregnancy, defined as insulin pump therapy integrated with CGM and an algorithm that automatically modulates insulin delivery in response to sensor glucose levels. Depending on the system, this may include automated basal adjustment, automated correction dosing or both. A system will be considered commercial if it was marketed for Type 1 diabetes at the time of study use or publication. Eligible evidence may therefore include off‐label pregnancy use and pregnancy‐specific clinical evaluation of a commercially available system. Newer systems undergoing pregnancy validation will be eligible only when the platform is commercially available for Type 1 diabetes and study results are available. Research‐only prototypes and non‐commercial algorithms will be excluded. Eligible systems may include MiniMed 670G/770G/780G, Tandem Control‐IQ, CamAPS FX, Omnipod 5 and related commercial algorithms. Regulatory indication, off‐label status, compatible pump and CGM configuration, algorithm target and jurisdiction at the time of the study will be extracted. Intrapartum outcomes will be extracted only when reported within studies evaluating AID as the primary pregnancy glucose‐management approach.

### 3.3 Comparator

Comparators will include sensor‐augmented pump therapy, defined as insulin pump therapy plus CGM without closed‐loop automation, including low‐glucose suspend or predictive low‐glucose suspend where reported; CGM‐supported multiple daily injections; and usual insulin therapy where clearly defined by study authors. Comparator categories will not be combined indiscriminately. Pairwise syntheses will be undertaken separately by comparator and study design. Single‐arm AID studies will be included for feasibility, acceptability, psychosocial, descriptive clinical and safety outcomes but will not contribute to comparative effect estimates or comparative certainty ratings.

### 3.4 Outcomes

#### 3.4.1 Primary Outcome

Percentage of time in the pregnancy‐specific target glucose range (3.5–7.8 mmol/L; 63–140 mg/dL), measured by CGM during pregnancy [8]. For the primary comparative meta‐analysis, second‐trimester estimates will be prioritised as a prespecified harmonisation rule. If second‐trimester data are unavailable, third‐trimester estimates and then first‐trimester estimates will be used. All trimester‐specific values will also be extracted and summarised separately, and trimester‐specific meta‐analyses will be undertaken where data permit.

#### 3.4.2 Secondary Outcomes

- Other CGM and glycaemic metrics will include time below range, time above range, overnight glycaemic metrics, glycaemic variability, coefficient of variation, glucose management index and HbA1c, where reported.

#### 3.4.3 Maternal Outcomes

- Maternal outcomes will include hypertensive disorders of pregnancy, including pre‐eclampsia; gestational weight gain; severe hypoglycaemia; diabetic ketoacidosis; progression of diabetic retinopathy; change in renal function or nephropathy; neuropathic complications where reported; mode of delivery; hospitalisation; and other serious maternal adverse events.

#### 3.4.4 Neonatal Outcomes

- Neonatal outcomes will include gestational age at delivery, preterm birth, birthweight and birthweight centiles, small‐for‐gestational‐age and large‐for‐gestational‐age birth, macrosomia, neonatal hypoglycaemia, neonatal hypoglycaemia requiring intravenous dextrose, neonatal intensive care admission, length of stay and perinatal mortality.

#### 3.4.5 Psychosocial Outcomes

- Psychosocial outcomes will include diabetes distress, fear of hypoglycaemia, treatment and device satisfaction, perceived burden, quality of life, sleep, anxiety, depression and eating‐disorder‐related outcomes where reported. For patient‐reported outcomes, the instrument name and version, construct, scoring range, direction of effect, validation status and assessment timepoint will be extracted. Pre‐existing mental health conditions and psychotropic medication use will be recorded as baseline characteristics where reported and considered as contextual or potential confounding factors rather than assumed causes of psychological outcomes.

#### 3.4.6 Feasibility and Implementation Outcomes

- Feasibility and implementation outcomes will include uptake and retention, time in closed‐loop or automation mode, discontinuation and reasons, technical problems, device‐related adverse events, clinician workload, consultation burden, training time and support requirements. Cost and access variables will include device and consumable costs, reimbursement or funding arrangements, patient out‐of‐pocket costs, staff and service resource use, health‐economic outcomes and reported access or equity considerations. These findings will be described in relation to jurisdiction, regulation, financing and model of antenatal diabetes care; causal effects on access, equity or workload will not be inferred unless supported by the study design.

#### 3.4.7 Postpartum Outcomes

- Early postpartum outcomes will be considered from delivery to 12 weeks postpartum. Where data permit, findings will be grouped as immediate inpatient postpartum (0–48 h), early post‐discharge (3–14 days) and later postpartum (6–12 weeks). Outcomes will include study‐defined and standard adult CGM metrics, total daily insulin dose, severe hypoglycaemia, diabetic ketoacidosis, breastfeeding‐related glycaemic events, hospital readmission, AID continuation or discontinuation, time in automation, technical problems, sleep, treatment burden, satisfaction and postpartum mental health measures.

### 3.5 Phenomena of Interest

The qualitative component will consider experiences, perceptions, acceptability, burden, confidence, safety concerns, clinician perspectives, implementation barriers and facilitators associated with AID use during T1D pregnancy.

### 3.6 Context

The review will consider antenatal, intrapartum and early postpartum contexts where AID is used as part of pregnancy diabetes care in outpatient, hospital, specialist diabetes, endocrinology or obstetric settings. Early postpartum is defined as delivery to 12 weeks postpartum. Intrapartum and postpartum findings will be synthesised separately from antenatal outcomes because glucose targets, insulin requirements and care settings differ.

### 3.7 Types of Studies

This review will consider quantitative, qualitative and mixed‐methods studies. Quantitative designs will include randomised controlled trials, non‐randomised comparative studies, prospective and retrospective cohorts, registries and case series. Qualitative designs will include interviews, focus groups, ethnographic, phenomenological, grounded theory and descriptive qualitative studies. Mixed‐methods studies will be included when quantitative or qualitative data can be clearly extracted. Exclusions include non‐original research (reviews, editorials and commentaries), conference abstracts without full reports and unpublished trials without available results. Conference abstracts will be excluded because they typically provide insufficient methodological detail and outcome data for appraisal. Trial registries and author contact will be used to identify completed studies and any corresponding full publications.

## 4 Methods

This protocol is reported in accordance with PRISMA‐P guidance for systematic review protocols [26]. The completed review will be reported in accordance with PRISMA 2020 [27]. Quantitative and qualitative evidence will be synthesised separately and integrated using a convergent segregated mixed‐methods approach, informed by established mixed‐methods evidence synthesis guidance [28]. The protocol is registered with PROSPERO (CRD420251025194).

### 4.1 Search Strategy

The search strategy will aim to identify published studies and trial registry records. The initial MEDLINE strategy was developed using controlled vocabulary and free‐text terms for Type 1 diabetes, pregnancy, AID, hybrid closed‐loop systems, artificial pancreas terminology and commercial device names. Every database and registry strategy will combine three concept blocks: Type 1 diabetes, pregnancy and AID/commercial systems, adapted to database‐specific indexing and syntax. Reference lists of included studies and related reviews will be screened, and forward citation tracking will be undertaken for key studies. Searches will cover 1 January 2010 onward because clinically relevant outpatient closed‐loop and commercial AID technologies emerged during this period; the start date has been broadened from 1 April to 1 January to avoid an arbitrary partial‐year restriction. Earlier research‐only prototypes are outside the commercial‐system eligibility criterion. No language restrictions will be applied. The database‐specific strategies are provided in File S1 and will be applied in the planned update search before final synthesis; execution dates, record counts and any resulting changes to study inclusion will be reported in the completed review.

### 4.2 Study Selection

Search results will be exported to EndNote for de‐duplication and then imported into Covidence for screening and data extraction. After pilot testing, two reviewers will independently screen titles and abstracts against the eligibility criteria. Full texts of potentially eligible studies will be assessed independently by two reviewers, with reasons for exclusion recorded. Before consensus resolution, raw percentage agreement and Cohen's kappa with 95% confidence intervals will be calculated and reported separately for title/abstract and full‐text screening. Disagreements will be resolved through discussion or consultation with a third reviewer. Study selection will be reported in a PRISMA flow diagram in the completed review [26].

### 4.3 Assessment of Methodological Quality

Two reviewers will independently assess methodological quality before synthesis using design‐specific critical appraisal tools appropriate to each study type. JBI critical appraisal instruments will be used for randomised trials, quasi‐experimental studies, cohort studies, case series and qualitative studies [29]. Disagreements will be resolved through discussion or consultation with a third reviewer. Appraisal findings will not be used as exclusion criteria but will inform interpretation, sensitivity analyses and certainty assessment where applicable [30, 31].

### 4.4 Data Extraction

Two reviewers will independently extract data using a piloted instrument adapted for this review (File S1). Quantitative extraction will capture study design, setting, population, baseline mental health conditions, intervention, comparator, commercial and regulatory status, device and algorithm, pregnancy‐specific target settings, timing of initiation, gestational and postpartum timepoints, automation use, outcome definitions, validated patient‐reported instruments, cost, reimbursement, resource use, results and risk‐of‐bias information. Qualitative extraction will capture study context, participants, sampling, data collection, analysis, findings, supporting quotations and implementation themes. The extraction form will be piloted on at least three studies and refined before full extraction. Authors will be contacted up to three times where data are missing or unclear.

### 4.5 Data Synthesis and Integration

The review will use a convergent segregated mixed‐methods design. Quantitative and qualitative evidence will be synthesised separately and then integrated through side‐by‐side comparison and mapping across outcome and implementation domains. Integration will be used to explain where quantitative findings are strengthened, limited or contextualised by qualitative evidence [28].

### 4.6 Quantitative Synthesis

Randomised and non‐randomised comparative studies will not be pooled together. Separate random‐effects meta‐analyses will be undertaken within study‐design and comparator strata when at least two studies are sufficiently similar in population, device, comparator, outcome definition and timepoint. For non‐randomised studies, adjusted effect estimates will be preferred and the confounders included in each model will be recorded. Continuous outcomes will be pooled as mean differences when instruments and scales are common, or standardised mean differences for conceptually equivalent validated instruments; dichotomous outcomes will be pooled as risk ratios with 95% confidence intervals. Rare outcomes, including diabetic ketoacidosis, severe hypoglycaemia and perinatal mortality, will use methods appropriate for sparse data and will be synthesised narratively when estimates are unstable. Comparators will be handled in separate pairwise syntheses: AID versus sensor‐augmented pump therapy, AID versus CGM‐supported multiple daily injections and AID versus clearly defined usual insulin therapy. If pairwise evidence is sparse, no cross‐comparator pooled estimate will be produced; structured narrative synthesis will be used. Network meta‐analysis is not planned a priori because device, population, timing and care‐model heterogeneity may violate transitivity. It will be considered only if the evidence forms a connected network, treatment nodes can be defined consistently, key effect modifiers are similarly distributed and statistical consistency can be assessed; any such analysis will be documented as a protocol amendment. For outcomes reported at multiple gestational timepoints, one estimate per study will be included in each meta‐analysis to avoid double counting. For pregnancy‐specific TIR, second‐trimester estimates will be prioritised; if unavailable, third‐trimester and then first‐trimester estimates will be used. All trimester‐specific values will also be summarised separately. Timing of AID initiation will be categorised as preconception, first trimester (< 14 weeks), second trimester (14–27 + 6 weeks) or third trimester (≥ 28 weeks). Where data permit, subgroup analyses will compare these categories and sensitivity analyses will restrict the evidence to initiation before conception or during the first trimester and exclude studies initiating AID after 14 weeks. Exposure duration will also be examined where reported. If multiple values are reported within the same trimester, the window closest to the end of that trimester, or the authors' late‐pregnancy window where boundaries are unclear, will be used and assumptions documented. For cross‐over randomised trials, first‐period data will be preferred to minimise carryover; paired estimates accounting for within‐participant correlation will otherwise be used when appropriate. Pregnancy will be the unit of analysis. Sensitivity analyses will exclude studies at high risk of bias and test outcome‐harmonisation assumptions. Heterogeneity will be assessed using *I* ^2^ and the chi‐square test. Where statistical or clinical heterogeneity is substantial, structured narrative synthesis will be prioritised. Device‐level estimates will not be interpreted as class effects when algorithms, glucose targets or pregnancy adaptation differ.

### 4.7 Qualitative Synthesis

Qualitative data will be synthesised using thematic synthesis following Thomas and Harden [32]. Relevant text, including participant quotations and author interpretations, will be coded to develop descriptive themes and higher‐order analytical themes related to user experience, psychological impact, confidence, burden, sleep, feasibility, clinician workload and health‐system factors [18, 23, 32].

### 4.8 Integration of Quantitative and Qualitative Evidence

Qualitative themes will be integrated with quantitative findings using structured side‐by‐side mapping. Themes will be linked to outcome domains where quantitative evidence is consistent, uncertain or heterogeneous and to implementation domains including acceptability, burden, usability, workflow, clinician workload, service readiness and patient‐, clinician‐ and service‐level barriers or facilitators [28, 32].

### 4.9 Reporting Bias and Certainty

Where at least 10 studies contribute to a meta‐analysis, funnel plots and appropriate tests for asymmetry will be considered. Trial registries and protocols will be compared with final publications to assess selective outcome reporting. Certainty of quantitative evidence will be assessed for prioritised comparative outcomes using GRADE [30]. Confidence in qualitative findings will be assessed using GRADE‐CERQual [31]. Single‐arm AID studies will not contribute to comparative certainty ratings but will inform feasibility, acceptability, implementation and safety‐signal interpretation.

## 5 Patient and Public Involvement

Patients and the public were not directly involved in the initial protocol design. Patient‐centred outcomes, including treatment burden, psychosocial impact, user experience, sleep, cost and access, were prespecified based on the literature and multidisciplinary clinical input [14, 18, 23]. Consumer and clinician input will be sought during interpretation of the completed synthesis, particularly when contextualising feasibility, acceptability, affordability, reported access differences and implementation findings. These contextual findings will not be interpreted causally unless supported by the underlying study design.

## 6 Amendments

Any important protocol amendments (changes to eligibility criteria, outcomes or analytic approach) will be documented in the final publication and, where appropriate, updated in the PROSPERO record.

## 7 Discussion

This protocol addresses a timely clinical and methodological question: how should the emerging evidence on commercial AID systems in Type 1 diabetes pregnancy be interpreted for clinical care, service planning and future research? Pregnancy remains a high‐risk and rapidly changing physiological state in which glycaemic targets are narrower than in non‐pregnant adults, insulin requirements change substantially across gestation and both hyperglycaemia and hypoglycaemia may have important maternal and neonatal consequences [2, 3, 4, 5, 7, 8, 9, 10, 11]. Although CGM has improved the measurement of glycaemic exposure and helped establish pregnancy‐specific time in range as a clinically meaningful outcome, insulin delivery in many current care models remains substantially dependent on user‐ and clinician‐directed adjustment [8, 9, 10, 11]. This creates a strong rationale for evaluating AID systems in pregnancy, while recognising that pregnancy‐specific use raises distinct clinical, regulatory and implementation questions.

The planned review is intentionally broader than a conventional efficacy review. In addition to pregnancy‐specific TIR and other CGM metrics, it will synthesise maternal and neonatal outcomes, diabetes‐specific adverse events, early postpartum outcomes, validated psychosocial measures, cost, reimbursement, resource use and implementation. This breadth is clinically necessary because the value of AID in pregnancy cannot be judged from glucose metrics alone. Translation into routine care also depends on device suitability, affordability, service capacity, training and support and user experience. Qualitative studies of women and clinicians will therefore be integrated with quantitative findings to explain heterogeneity and identify reported barriers and enablers without attributing causality beyond the supporting evidence.

A central feature of this protocol is its attention to heterogeneity. Commercial AID systems differ in algorithm design, glucose target settings, automated correction capability and pregnancy‐specific adaptation [12, 13, 14]. Studies also differ in timing of AID initiation, comparator regimen, baseline technology experience, clinical support model, gestational timepoint and outcome definition [14, 15, 16, 17, 20, 21, 22]. These differences are not simply a methodological inconvenience; they are clinically meaningful. For this reason, pooled estimates will be interpreted cautiously and meta‐analysis will be undertaken only where studies are sufficiently comparable in population, intervention, comparator and outcome definition. Where pooling is inappropriate, structured narrative synthesis will be prioritised. This approach is intended to avoid treating commercial AID as a single uniform intervention when device‐level and care‐model differences may influence outcomes.

Several limitations are anticipated. First, the available evidence is likely to include a small number of randomised trials, observational cohorts and case series, limiting certainty for less frequent maternal and neonatal outcomes. Second, outcome definitions and reporting windows may vary across studies, particularly for neonatal hypoglycaemia, device‐related adverse events, psychosocial outcomes, automation exposure and implementation measures. Third, some studies may evaluate systems used off‐label or with non‐pregnancy‐specific settings, which may limit generalisability across regulatory environments and health systems. Fourth, qualitative evidence may come predominantly from specialist centres or early‐adopter services, potentially under‐representing settings with less technology access, fewer trained staff or different models of antenatal diabetes care. These limitations will be addressed through design‐specific critical appraisal, transparent reporting of harmonisation assumptions, sensitivity analyses where feasible, cautious interpretation of pooled estimates and integration of qualitative evidence to contextualise feasibility, acceptability, clinician workload and service readiness.

The review will also have important strengths. It is prospectively registered, aligned with PRISMA‐P and designed to include randomised, non‐randomised, observational, qualitative and mixed‐methods evidence [26, 27, 28, 32]. The use of GRADE and GRADE‐CERQual will allow quantitative certainty and confidence in qualitative findings to be assessed separately, while the convergent segregated approach will support integration across clinical and implementation domains [28, 30, 31]. This is particularly suited to AID in pregnancy, where evidence from trials, real‐world cohorts and qualitative studies each answers a different but complementary question.

The completed review should provide clinicians, researchers, guideline developers and health services with a clearer account of what is currently known about commercial AID use in Type 1 diabetes pregnancy. It should identify where evidence is sufficiently consistent to inform practice, where uncertainty remains and which patient‐, device‐, clinician‐ and service‐level factors require further evaluation. By integrating effectiveness, safety, psychosocial and implementation evidence, the review aims to support a more clinically useful interpretation of AID in pregnancy: not only whether these systems improve outcomes, but for whom, under what conditions and with what practical requirements for safe and equitable care.

## Author Contributions

**Elahe Sheklabadi:** data curation, formal analysis, validation, writing – review and editing. **Jieli Li:** writing – original draft, writing – review and editing, data curation, formal analysis. **Rebecca F. Goldstein:** writing – review and editing, methodology, conceptualization, validation, supervision. **Ashley H. Ng:** conceptualization, methodology, supervision, formal analysis, writing – review and editing, data curation, validation. **Helena Teede:** methodology, supervision, writing – review and editing. **Negar Naderpoor:** conceptualization, methodology, software, data curation, supervision, resources, formal analysis, project administration, validation, investigation, writing – review and editing, writing – original draft.

## Funding

The authors have nothing to report.

## Ethics Statement

Ethics approval and consent to participate are not applicable, as this article describes a systematic review protocol and does not involve recruitment of participants or collection of primary participant data.

## Consent

The authors have nothing to report.

## Conflicts of Interest

The authors declare no conflicts of interest.

## Supporting information


**File S1:** Endocrinology, diabetes & metabolism.
**Table S1:** MEDLINE (Ovid) search strategy. Search date: 30 December 2025.
**Table S2:** Emcare (Ovid) search strategy. Search date: 30 December 2025.
**Table S3:** Embase (Ovid) search strategy. Search date: 30 December 2025.
**Table S4:** CINAHL (EBSCOhost) revised search strategy. Execution details: to be reported in the completed review.
**Table S5:** Scopus revised search strategy. Execution details: to be reported in the completed review.
**Table S6:** ClinicalTrials.gov revised advanced search strategy. Execution details: to be reported in the completed review.
**Table S7:** Cochrane Library revised search strategy. Execution details: to be reported in the completed review.
**Table S8:** Cochrane Central Register of Controlled Trials search strategy. Search date: 30 December 2025.
**Table S9:** Planned data extraction domains.

## Data Availability

No new datasets were generated or analysed for this protocol. Data extracted for the completed systematic review and analytic code will be made available where feasible, subject to copyright restrictions relating to published source materials.
