# Supplementary material for: Commercial Automated Insulin Delivery Systems in Type 1 Diabetes Pregnancy: Protocol for a Mixed‐Methods Systematic Review and Meta‐Analysis of Glycaemic, Perinatal, Psychosocial and Implementation Outcomes
Source: Endocrinol Diabetes Metab. 2026 Sep 5;9(5):e70309. doi: 10.1002/edm2.70309 (PMC13546029; doi:10.1002/edm2.70309)
Supplement: Supplementary file 1 [file EDM2-9-e70309-s001.docx]

**Supplementary File 1 – Endocrinology, Diabetes & Metabolism**

**Search strategies and planned data extraction instrument**

This supplementary file accompanies the manuscript entitled “Commercial automated insulin delivery systems in type 1 diabetes pregnancy: protocol for a mixed-methods systematic review and meta-analysis of glycaemic, perinatal, psychosocial and implementation outcomes.” All database and registry strategies combine three concept blocks: type 1 diabetes, pregnancy and automated insulin delivery/commercial systems. The corrected strategies are prespecified here and will be applied in the planned update search before final synthesis; execution dates, record counts and any resulting changes to study inclusion will be reported in the completed review. The data extraction instrument will be piloted before full extraction and refined if required, with substantive amendments documented in the completed review.

# **Search strategies**

Search strategies include controlled vocabulary where available, free-text synonyms and commercial device/platform names to improve sensitivity. Database-specific syntax is used for type 1 diabetes, pregnancy and automated insulin delivery, including CamAPS FX, MiniMed 670G/770G/780G, Tandem Control-IQ, Omnipod 5, DBLG1 and related commercial algorithms. Searches cover 1 January 2010 onward and will be updated immediately before the final synthesis.

## **Supplementary Table S1. MEDLINE (Ovid) search strategy. Search date: 30 December 2025**

| **Search line** | **Query** | **Records retrieved** |
| --- | --- | --- |
| #1 | Diabetes Type one OR Type one Diabetes mellitus OR Type 1 Diabetes OR T1D OR Insulin-dependent diabetes mellitus.mp. | 73488 |
| #2 | exp Diabetes Mellitus, Type 1/ OR exp Type 1 diabetes/ | 90976 |
| #3 | #1 OR #2 | 116831 |
| #4 | exp Pregnancy/ OR exp Pregnancy, High-Risk/ OR exp Maternal Health/ | 1057447 |
| #5 | Pregnan* OR Antenatal OR perinatal OR Expectant mothers OR Maternal.mp. | 1362256 |
| #6 | #4 OR #5 | 1375847 |
| #7 | #3 AND #6 | 6163 |
| #8 | Hybrid closed-loop OR hybrid closed loop OR Automated insulin delivery OR Artificial pancreas OR Closed-loop insulin therapy OR closed loop OR CamAPS FX OR MiniMed 670G OR MiniMed 770G OR MiniMed 780G OR Control-IQ OR Tandem t:slim X2 OR Omnipod 5 OR DBLG1.mp. | 2845 |
| #9 | Hybrid insulin pump OR Hybrid closed loop pump OR Automated insulin pump.mp. | 18 |
| #10 | Sensor-augmented insulin pump OR Continuous subcutaneous insulin infusion.mp. | 2523 |
| #11 | Insulin pump therapy OR Continuous glucose monitoring OR Multiple daily injections OR insulin therapy.mp. | 22432 |
| #12 | exp Insulin Infusion Systems/ OR exp Artificial Pancreas/ OR exp Continuous Glucose Monitoring/ OR exp insulin pump/ OR exp insulin therapy/ | 7959 |
| #13 | #8 OR #9 OR #10 OR #11 OR #12 | 28381 |
| #14 | #7 AND #13 | 792 |
| #15 | limit #14 to yr="2010 - 2025" | 539 |

**Supplementary Table S2. Emcare (Ovid) search strategy. Search date: 30 December 2025**

| **Search line** | **Query** | **Records retrieved** |
| --- | --- | --- |
| #1 | Diabetes Type one OR Type one Diabetes mellitus OR Type 1 Diabetes OR T1D OR Insulin-dependent diabetes mellitus.mp. | 139177 |
| #2 | Pregnan* OR Antenatal OR perinatal OR Expectant mothers OR Maternal.mp. | 381211 |
| #3 | Hybrid closed-loop OR hybrid closed loop OR Automated insulin delivery OR Artificial pancreas OR Closed-loop insulin therapy OR closed loop OR CamAPS FX OR MiniMed 670G OR MiniMed 770G OR MiniMed 780G OR Control-IQ OR Tandem t:slim X2 OR Omnipod 5 OR DBLG1.mp. | 1946 |
| #4 | Hybrid insulin pump OR Hybrid closed loop pump OR Automated insulin pump.mp. | 18 |
| #5 | Sensor-augmented insulin pump OR Continuous subcutaneous insulin infusion.mp. | 1292 |
| #6 | Insulin pump therapy OR Continuous glucose monitoring OR Multiple daily injections OR insulin therapy.mp. | 12010 |
| #7 | #1 AND #2 | 6231 |
| #8 | #3 OR #4 OR #5 OR #6 | 13425 |
| #9 | #7 AND #8 | 511 |
| #10 | limit #9 to yr="2010 - 2025" | 441 |

## **Supplementary Table S3. Embase (Ovid) search strategy. Search date: 30 December 2025**

| **Search line** | **Query** | **Records retrieved** |
| --- | --- | --- |
| #1 | Diabetes Type one OR Type one Diabetes mellitus OR Type 1 Diabetes OR T1D OR Insulin-dependent diabetes mellitus.mp. | 523765 |
| #2 | exp Diabetes Mellitus, Type 1/ OR exp Type 1 diabetes/ | 174115 |
| #3 | #1 OR #2 | 534560 |
| #4 | exp Pregnancy/ OR exp Pregnancy, High-Risk/ OR exp Maternal Health/ | 1005591 |
| #5 | Pregnan* OR Antenatal OR perinatal OR Expectant mothers OR Maternal.mp. | 1675453 |
| #6 | #4 OR #5 | 1675870 |
| #7 | #3 AND #6 | 19761 |
| #8 | Hybrid closed-loop OR hybrid closed loop OR Automated insulin delivery OR Artificial pancreas OR Closed-loop insulin therapy OR closed loop OR CamAPS FX OR MiniMed 670G OR MiniMed 770G OR MiniMed 780G OR Control-IQ OR Tandem t:slim X2 OR Omnipod 5 OR DBLG1.mp. | 5950 |
| #9 | Hybrid insulin pump OR Hybrid closed loop pump OR Automated insulin pump.mp. | 40 |
| #10 | Sensor-augmented insulin pump OR Continuous subcutaneous insulin infusion.mp. | 4415 |
| #11 | Insulin pump therapy OR Continuous glucose monitoring OR Multiple daily injections OR insulin therapy.mp. | 41815 |
| #12 | exp Insulin Infusion Systems/ OR exp Artificial Pancreas/ OR exp Continuous Glucose Monitoring/ OR exp insulin pump/ OR exp insulin therapy/ | 62257 |
| #13 | #8 OR #9 OR #10 OR #11 OR #12 | 81231 |
| #14 | #7 AND #13 | 2733 |
| #15 | limit #14 to yr="2010 - 2025" | 2238 |

## **Supplementary Table S4. CINAHL (EBSCOhost) revised search strategy. Execution details: to be reported in the completed review**

| **Search line** | **Query** | **Records retrieved** |
| --- | --- | --- |
| #1 | (MH "Diabetes Mellitus, Type 1+") OR TX ("type 1 diabetes" OR "type 1 diabetes mellitus" OR T1D OR "insulin-dependent diabetes mellitus") | Pending |
| #2 | (MH "Pregnancy+") OR (MH "Pregnancy in Diabetics") OR TX (pregnan* OR antenatal OR perinatal OR maternal OR "expectant mother*") | Pending |
| #3 | TX ("hybrid closed-loop" OR "hybrid closed loop" OR "automated insulin delivery" OR "artificial pancreas" OR "closed-loop insulin therapy" OR "closed loop" OR "CamAPS FX" OR "MiniMed 670G" OR "MiniMed 770G" OR "MiniMed 780G" OR "Control-IQ" OR "Tandem t:slim X2" OR "Omnipod 5" OR DBLG1) | Pending |
| #4 | #1 AND #2 AND #3 | Pending |
| #5 | Limit #4 to publication years 2010-present | Pending |

## **Supplementary Table S5. Scopus revised search strategy. Execution details: to be reported in the completed review**

| **Search line** | **Query** | **Records retrieved** |
| --- | --- | --- |
| #1 | TITLE-ABS-KEY ( ("type 1 diabetes" OR "type 1 diabetes mellitus" OR T1D OR "insulin-dependent diabetes mellitus") AND (pregnan* OR antenatal OR perinatal OR maternal) AND ("hybrid closed-loop" OR "hybrid closed loop" OR "automated insulin delivery" OR "artificial pancreas" OR "closed-loop insulin therapy" OR "closed loop" OR "CamAPS FX" OR "MiniMed 670G" OR "MiniMed 770G" OR "MiniMed 780G" OR "Control-IQ" OR "Tandem t:slim X2" OR "Omnipod 5" OR DBLG1) ) AND PUBYEAR > 2009 | Pending |

## **Supplementary Table S6. ClinicalTrials.gov revised advanced search strategy. Execution details: to be reported in the completed review**

| **Search line** | **Query** | **Records retrieved** |
| --- | --- | --- |
| #1 | Condition or disease field: "type 1 diabetes" OR "type 1 diabetes mellitus" OR T1D | Pending |
| #2 | Other terms field: pregnancy OR pregnant OR antenatal OR maternal OR perinatal | Pending |
| #3 | Intervention/treatment field: "automated insulin delivery" OR "hybrid closed loop" OR "hybrid closed-loop" OR "artificial pancreas" OR "CamAPS FX" OR "MiniMed 670G" OR "MiniMed 770G" OR "MiniMed 780G" OR "Control-IQ" OR "Tandem t:slim X2" OR "Omnipod 5" OR DBLG1 | Pending |
| #4 | Apply #1 AND #2 AND #3 using the ClinicalTrials.gov advanced-search fields; include studies first posted or commencing from 1 January 2010 onward | Pending |

## **Supplementary Table S7. Cochrane Library revised search strategy. Execution details: to be reported in the completed review**

| **Search line** | **Query** | **Records retrieved** |
| --- | --- | --- |
| #1 | MeSH descriptor: [Diabetes Mellitus, Type 1] explode all trees | Pending |
| #2 | ("type 1 diabetes" OR "type 1 diabetes mellitus" OR T1D OR "insulin-dependent diabetes mellitus"):ti,ab,kw | Pending |
| #3 | MeSH descriptor: [Pregnancy] explode all trees | Pending |
| #4 | (pregnan* OR antenatal OR perinatal OR maternal OR "expectant mother*"):ti,ab,kw | Pending |
| #5 | ("hybrid closed-loop" OR "hybrid closed loop" OR "automated insulin delivery" OR "artificial pancreas" OR "closed-loop insulin therapy" OR "closed loop" OR "CamAPS FX" OR "MiniMed 670G" OR "MiniMed 770G" OR "MiniMed 780G" OR "Control-IQ" OR "Tandem t:slim X2" OR "Omnipod 5" OR DBLG1):ti,ab,kw | Pending |
| #6 | (#1 OR #2) AND (#3 OR #4) AND #5 | Pending |
| #7 | Limit #6 to publication years 2010-present | Pending |

## **Supplementary Table S8. Cochrane Central Register of Controlled Trials search strategy. Search date: 30 December 2025**

| **Search line** | **Query** | **Records retrieved** |
| --- | --- | --- |
| #1 | Diabetes Type one OR Type one Diabetes mellitus OR Type 1 Diabetes OR T1D OR Insulin-dependent diabetes mellitus.mp. | 32443 |
| #2 | Pregnan* OR Antenatal OR perinatal OR Expectant mothers OR Maternal.mp. | 102672 |
| #3 | Hybrid closed-loop OR hybrid closed loop OR Automated insulin delivery OR Artificial pancreas OR Closed-loop insulin therapy OR closed loop OR CamAPS FX OR MiniMed 670G OR MiniMed 770G OR MiniMed 780G OR Control-IQ OR Tandem t:slim X2 OR Omnipod 5 OR DBLG1.mp. | 855 |
| #4 | Hybrid insulin pump OR Hybrid closed loop pump OR Automated insulin pump.mp. | 9 |
| #5 | Sensor-augmented insulin pump OR Continuous subcutaneous insulin infusion.mp. | 877 |
| #6 | Insulin pump therapy OR Continuous glucose monitoring OR Multiple daily injections OR insulin therapy.mp. | 7188 |
| #7 | exp Diabetes Mellitus, Type 1/ OR exp Type 1 diabetes/ | 7612 |
| #8 | #1 OR #7 | 34340 |
| #9 | exp Pregnancy/ OR exp Pregnancy, High-Risk/ OR exp Maternal Health/ | 32640 |
| #10 | #2 OR #9 | 102936 |
| #11 | #8 AND #10 | 1033 |
| #12 | exp Insulin Infusion Systems/ OR exp Artificial Pancreas/ OR exp Continuous Glucose Monitoring/ OR exp insulin pump/ OR exp insulin therapy/ | 1021 |
| #13 | #3 OR #4 OR #5 OR #6 OR #12 | 8344 |
| #14 | #11 AND #13 | 243 |
| #15 | limit #14 to yr="2010 - 2025" | 216 |

# **Planned data extraction instrument**

The extraction instrument will be piloted independently by two reviewers on at least three included studies and refined before full extraction. Extracted fields will be recorded in Covidence and/or a structured spreadsheet. The instrument distinguishes randomised, non-randomised comparative, single-arm and qualitative evidence; records timing of AID initiation and regulatory context; and captures validated psychosocial instruments, cost, resource use and outcomes through 12 weeks postpartum.

## **Supplementary Table S9. Planned data extraction domains**

| **Domain** | **Variables to extract** |
| --- | --- |
| Study identification | Author; year; title; journal; country; funding; conflicts; registration/protocol; linked reports; publication status; duplicate or overlapping cohorts |
| Design and setting | Study design; recruitment period; care setting; number of centres; country/health system; model of antenatal diabetes care; inclusion/exclusion criteria; randomised, non-randomised comparative, single-arm, qualitative or mixed-methods classification |
| Participants | Sample size (AID); sample size (control); type 1 diabetes duration; age; parity; ethnicity; socioeconomic indicators; BMI; baseline HbA1c; baseline CGM/pump use; gestational age at enrolment; pre-existing depression, anxiety, eating disorder or other mental health condition; psychotropic medication use where reported |
| Intervention | Commercial system and configuration; pump and CGM components; algorithm and automated correction capability; pregnancy-specific target settings; commercial availability at time of study; pregnancy indication and regulatory jurisdiction; off-label status; timing of initiation (preconception, <14 weeks, 14-27+6 weeks, >=28 weeks); training/support; automation use; duration of exposure; intrapartum and postpartum continuation |
| Comparator | Sensor-augmented pump therapy; CGM-supported multiple daily injections; usual care; sensor type; pump type; insulin regimen; low-glucose suspend/predictive low-glucose suspend; education and clinical-support model; comparator definition and treatment crossover |
| CGM and glycaemic outcomes | Pregnancy TIR 63-140 mg/dL; TBR <63 and <54 mg/dL; TAR >140, >180 and >250 mg/dL; mean glucose; coefficient of variation; glucose management indicator; HbA1c; trimester-specific timepoints; overnight outcomes; postpartum study-defined targets and adult TIR 70-180 mg/dL where reported; total daily insulin dose |
| Maternal outcomes | Severe hypoglycaemia; diabetic ketoacidosis; hypertensive disorders; gestational weight gain; progression of diabetic retinopathy; renal function/nephropathy; neuropathic complications; mode of delivery; hospitalisation/readmission; adverse events |
| Neonatal outcomes | Gestational age; preterm birth; birthweight; birthweight centile; large-for-gestational-age/small-for-gestational-age birth; macrosomia; neonatal hypoglycaemia; intravenous dextrose; NICU admission; NICU length of stay; perinatal mortality |
| Feasibility and implementation | Uptake; retention; time in closed loop/automation; discontinuation and reasons; device issues; clinician workload; consultation burden; training time and needs; service staffing; device and consumable costs; reimbursement/funding; patient out-of-pocket costs; resource use; health-economic outcomes; reported access and equity considerations; regulatory and service context |
| Psychosocial outcomes | Fear of hypoglycaemia; diabetes distress; treatment/device satisfaction; quality of life; sleep; perceived burden; anxiety; depression; eating-disorder-related outcomes; instrument name/version; construct; scoring range; direction; validation status; assessment timepoint; qualitative experience |
| Qualitative evidence | Sampling; data collection; analysis method; participant quotations; author themes; credibility/illustrations; patient, clinician, device, financing, regulatory and service-level barriers/enablers |
| Risk of bias/appraisal | Design-specific JBI appraisal responses; qualitative appraisal; unresolved concerns; impact on synthesis |
| Analysis-ready data | Effect estimates; SE/SD/CI; denominators; adjusted and unadjusted estimates; covariates in adjusted models; randomised/non-randomised stratum; comparator stratum; conversion and harmonisation assumptions; missing data; author contact and clarification status |
| Reviewer notes | Extraction reviewer; verification reviewer; disagreements; resolution; amendments to extraction form; screening agreement and Cohen's kappa recorded separately for title/abstract and full-text stages |
| Postpartum outcomes | Window from delivery to 12 weeks postpartum; immediate inpatient (0-48 h), early post-discharge (3-14 d) and later postpartum (6-12 wk) where available; CGM metrics; insulin dose; severe hypoglycaemia; diabetic ketoacidosis; breastfeeding-related glycaemic events; readmission; AID continuation; time in automation; device problems; sleep; burden; satisfaction; anxiety and depression |
